# Supplementary material for: Measurement of symphysis fundal height for gestational age estimation in low-to-middle-income countries: A systematic review and meta-analysis
Source: PLoS One. 2022 Aug 25;17(8):e0272718. doi: 10.1371/journal.pone.0272718 (PMC9409500; doi:10.1371/journal.pone.0272718)
Supplement: S1 Appendix — (DOCX) [file pone.0272718.s002.docx]

**Measurement of symphysis fundal height for gestational age estimation in low-to-middle-income countries: A systematic review and meta-analysis**

**S1 Supplementary Appendix**

**Table of Contents**

[**S1 Text. Search Strategy 2**](#_heading=h.2s8eyo1)

**S2** [**Text. Variables for Data Extraction 4**](#_heading=h.17dp8vu)

[**S3 Text. QUADAS-2 Assessment Criteria 5**](#_heading=h.3rdcrjn)

[**S1 Table. Basic Description of Included and Excluded Studies 6**](#_heading=h.3znysh7)

[**S2 Table. Methods for Converting or Calculating Gestational Age from Symphysis Fundal Height 9**](#_heading=h.26in1rg)

**S**[**3 Table. Population-based reference data of SFH measurements by LMP/BOE estimated gestational age in LMICs, by region 10**](#_heading=h.lnxbz9)

**S4 Table. LMP-based studies reporting upon the accuracy of Symphysis Fundal Height to estimate Gestational Age 11**

[**S5 Table. Inter-rater and Intra-rater Reliability Assessments 12**](#_heading=h.35nkun2)

[**S1 Fig. QUADAS-2 summary for studies reporting agreement between SFH and gold standard GA 14**](#_heading=h.tyjcwt)

[**S1 Appendix References 15**](#_heading=h.4d34og8)

**S1 Text. Search Strategy**

**Dates of Searches**

1. April 14, 2015
2. January 20, 2018
3. May 20, 2020
4. November 17, 2021

**Search terms**

(“uterine height”[TIAB] OR “uterine fundus”[TIAB] OR “fundus uteri”[TIAB] OR “fundus height”[TIAB] OR “symphysis-fundus”[TIAB] OR “symphysis fundus”[TIAB] OR “symphyseal”[TIAB] OR “symphysial”[TIAB] OR SFH[TIAB] OR “symphysis-fundus distance”[TIAB] OR “symphysis fundus distance”[TIAB] OR “symphysio-fundal height”[TIAB] OR “symphysio fundal height”[TIAB] OR “symphysis to fundal height”[TIAB] OR “symphysis to fundus”[TIAB] OR “symphysis-pubis”[TIAB] OR “symphysis pubis”[TIAB] OR “pubic symphyseal height”[TIAB] OR “fundal height”[TIAB] OR “symphysiofundal”[TIAB]) OR (symphy*[TIAB] AND ("fundal"[TIAB] OR "fundus"[TIAB] OR "pubis"[TIAB] OR "pubic"[TIAB] OR "height"[TIAB] OR "distance”[TIAB] OR "fundal"[TIAB] OR "fundus"[TIAB])) AND (“Gestational Age"[MeSH Terms] OR “gestational age”[All Fields] OR “menstrual age”[All Fields]) AND "humans"[MeSH Terms]

**Databases:**

- PubMed
- Embase
- Cochrane
- Web of Science
- Popline
- Global Health Libraries

**Inclusion Criteria:**

- Data comparing SFH and gestational age: Agreement/accuracy data for dating pregnancies, OR normative growth curves derived from population data (tables, curves, equations)
  - Agreement or accuracy: LMICs or HICs
  - Normative: LMIC ONLY
- Data provided on inter or intra rater reliability
- Gold standard gestational age is an antenatal method (e.g., ultrasound, best obstetric estimate, or LMP)
- Representative of the general obstetric population, not highly selective populations (i.e. the study cohort reflects the majority of general obstetric population; at least ⅔)
  - ***except for Inter-rater or Intra-rater reliability assessments of SFH measurements***

**Exclusion Criteria:**

- Selected populations (not representing the general obstetric population: eg. Caucasians living in China, HIV+ population, twin studies, only optimally health populations that systematically excluded maternal or pregnancy morbidities)
  - except for Inter-rater or Intra-rater reliability assessments of SFH measurements
- <50 patients
- Gold standard for gestational age dating is clinical newborn exam or birthweight or time of delivery
- Cannot translate with available tools (Google translate)
- Could not track down paper after extensive library search, librarian assistance, contacting corresponding author
- Published before 1980 (except for review of SFH techniques)
- Editorials, reviews, or reports with no primary data

***Exclusion for particular Objectives***

- Normal growth: High income countries excluded
- SFH agreement with reference standard:
  - Only for determining infant size (i.e. small-for-gestational-age, appropriate-for-gestational-age, large-for-gestational-age, or to determine estimated fetal weight)
  - Excluded small-for-gestational age babies

**S2 Text. Variables for Data Extraction**

| **Study characteristics** | **Reference Standard Assessment Methodology** | **SFH Assessment Methodology** | **Normative curve data** | **Agreement between SFH and reference standard** | **Diagnostic Accuracy for preterm (<34, <36, or <37 weeks)** |
| --- | --- | --- | --- | --- | --- |
| - Author - Journal - Year of publication - City, district, country - Study design - Clinical setting - Population characteristics - Sampling method - Eligibility criteria - Sample size - Sample characteristics - Study period | - Description of reference standard measurement method - If ultrasound, parameters and reference curve used - Timing of assessment during pregnancy - Type of health worker performing the assessment - Mean and SD of GA at enrollment | - Description of measurement method - Type of health worker performing the assessment - Whether the health worker was blinded to reference standard GA and/or measurement scale - Timing of measurement during pregnancy - Number of repeat measures within visit - Number of repeat measures within pregnancy - Inter- and intra-rater reliability statistics - Mean and SD SFH measurement - Mean and SD GA by SFH | - Methodology for creating curve - Regression equation; R^2^ - Mean or median SFH by week in cm - Sd for SFH by week in cm - Sample size for SFH by week | - Pearson correlation coefficient - Concordance correlation coefficient - Intraclass correlation coefficient - Mean difference and SD - Prediction error - 95% prediction CI (limits of agreement or prediction interval) - Trend of Bland-Altman plot - % within 1 week of reference standard GA - % within 2 weeks of reference standard GA | - Total number and % of preterm by reference standard - Total number and % of preterm by SFH - Kappa statistic - ROC-AUC - Sensitivity (95% CI) - Specificity (95% CI) - Positive predictive value - Negative predictive value |

**S3 Text. QUADAS-2 Assessment Criteria**

| **Domain 1: Patient Selection** | **Domain 2: Test Method** | **Domain 3: Gold Standard** | **Domain 4: Flow and Timing** |
| --- | --- | --- | --- |
| - Was the sampling methodology clearly reported? - Were participants randomly or consecutively reported? - Did the study avoid inappropriate exclusion criteria? | - Was the test method clearly described? - Was the test method measured >1 time per reader? - Were quality control measures used and reported? - Was training the measurers to use the test method described? - Were the test method results interpreted without knowledge of the results of the gold standard? - Was a pre-set threshold used to determine prematurity? | - Was the gold standard method stated as such? - Was the gold standard method clearly described? - Is the gold standard likely to correctly classify gestational age? - Were the gold standard results interpreted without knowledge of the results of the test method? | - Were all participants included in receiving the test method? - Were all patients measured by the gold standard? - Did all patients receive the same gold standard? - Were all participants included in the analysis? |

**S1 Table. Basic Description of Included and Excluded Studies**

| **Author (Year)** | **Location**  Country (district, town) | **Study Setting** | **Study Design** | **Sample Size** | **Gold Standard Method of GA Determination** | **Method and Timing of SFH measurement** |
| --- | --- | --- | --- | --- | --- | --- |
| **African LMIC Studies** | | | | | | |
| Adewale (2011)^1^ | Nigeria (Ilorian, Kwara) | Tertiary Hospital ANC | Prospective longitudinal | 390 | LMP | 20-40 weeks; Belizan technique |
| Amagloh (2009)^2^ | Ghana (Kassena-Nankana District and Bolgatanga Municipal Assembly in the Upper East Region) | Tertiary Hospital | Cross-sectional | 100 | LMP (Not clearly stated) | 1^st^, 2^nd^, 3^rd^ trimesters |
| Challis (2002)^3^ | Mozambique (Maputo City; *suburban, semi-rural areas*) | ANC Clinics | Prospective longitudinal | 817 (6544) | BOE (LMP & Ultrasound); (14-21 weeks) | 15-42 weeks; Westin technique |
| Ebite (2009)^4^ | Nigeria (Uromi, Edo Central district; *97% from Esan tribe*) | Tertiary Hospital | Prospective longitudinal | 202 | LMP (& Ultrasound if have irregular periods) | 20-40 weeks |
| Kiserud (1986)^5^ | Ethiopia (Arba Minch; *excluded preterm infants*) | Primary Health Center free/ low cost ANC | Prospective | 114 (500) | Ultrasound (<22 weeks) | 16-43 weeks; Westin |
| Mador (2011)^6^ | Nigeria (Jos) | Tertiary Hospital ANC | Cross-sectional | 405 | Ultrasound (timing not stated) | 14-40 weeks |
| Malaba (2018)^7^ | South Africa (Cape Town; *low SES area, 43% HIV+*) | Primary Health Clinic/ peripheral facility | Prospective | 732 | Ultrasound (≤24 weeks) | ≤24 weeks |
| Ogunranti (1990)^8^ | Nigeria (Port Harcourt) | Tertiary Hospital (hospitals and maternity homes) | Retrospective cross-sectional | 581 | LMP (& date of birth) | 20-40 weeks |
| Rada (2018)^9^ | Benin, Gabon, Mozambique, & Tanzania | Not clearly stated | Retrospective chart review (patients prospectively recruited for a RCT) | 3038 | LMP | $\geq$20 weeks |
| Sogbanmu (1980)^10^ | Nigeria (Ondo State) | Tertiary Hospital ANC | Prospective longitudinal | 159 | LMP (Not clearly stated) | $\geq$9 weeks |
| van Bogaert (1999)^11^ | South Africa (Eastern Cape Province; *Xhosa women*) | Tertiary Hospital ANC | Cross-sectional | 800 | Ultrasound (average 24 weeks) | 12-40 weeks |
| van Rensburg (2003)^12^ | South Africa (Bloemfontein, Mangaung) | Primary Health Center | Cross-sectional | 208 | Ultrasound (<24 weeks) | 12-40 weeks |
| **Asian LMIC Studies** | | | | | | |
| Agarwal (2002)^13^ | India (Varnasi, Uttar Pradesh) | Community based | Prospective longitudinal | 3700 (11100) | LMP (Not clearly stated) | 14-18, 26-30, and 34-38 weeks |
| Deeluea (2013)^14^ | Thailand (northern) | Tertiary Hospital | Retrospective time-series | 1038 (7523)  648 LMP, 390 U | LMP or Ultrasound  (average 16 weeks) | 20-40 weeks |
| Fikree (1988)^15^ | Pakistan (Karachi; *low SES urban squatter settlements*) | community based survey | Cross-sectional | 163 | LMP | 1^st^, 2^nd^, 3^rd^ trimesters |
| Ghate (1996)^16^ | India (Mangalwar Peth & Sadashev Peth) | Tertiary Hospitals | Prospective | 281 (281) | LMP | 26-40 weeks |
| Indira (1990)^17^ | India (Pondicherry) | ANC Clinic | Prospective | 109 (706) | LMP | $\geq$13 weeks |
| Jehan (2010)^18^ | Pakistan (Hyderabad) | Community Based | Prospective longitudinal, population-based cohort | 1128 | Ultrasound (20-26 weeks) | 20-26 weeks; Westin |
| Karl (2015)^19^ | Papua New Guinea (Madang, North PNG) | Primary Health Center | Prospective (enrolled in parent RCT) | 688 & 502 | Ultrasound (6-<25) | $\geq$6 weeks |
| Lee (2020)^20^ | Bangladesh (Sylhet; *rural*) | Community-based | Prospective longitudinal | 1486 (3414) & 748 (2244) (for norms: 1146 (3480)) | Ultrasound (<20 weeks) | 24-28, 32-36, and >37 weeks |
| Limpanyalert & Manotaya (2001)^21^ | Thailand (Bangkok) | Tertiary Hospital | Prospective longitudinal | 199 (879) | BOE (LMP & Ultrasound) (<12 weeks) | 16-40 weeks; Westin |
| Mathai (1987)^22^ | India (Vellore) | Tertiary Hospital ANC | Prospective | 250 (584) | LMP (Not clearly stated) | 20-40 weeks |
| Moore (2015)^23^ | Thailand (Maela Refugee Camp, Thai-Burma border) | Primary Health Clinic/ peripheral facility | Retrospective chart review | 704 | Ultrasound (7-<14 weeks) | $\geq$16 weeks |
| Rao (2014)^24^ *^conference poster^* | India (Sullia, Karnataka) | Tertiary Hospital | Prospective | 100 (400) | BOE (LMP & Ultrasound) | 12-42 weeks |
| Shamawarna (2012)^25^ | Sri Lanka (Galle) | Tertiary Hospital ANC | Prospective | 400 (800) | Ultrasound (<22 weeks) | 20-41 weeks |
| Shrestha (2017)^26^ | Nepal (Banke district) | Not clearly stated | Prospective | 614 | Ultrasound (18-26 weeks) | 18-26 weeks |
| Ulstein (1988)^27^ | Nepal (Kathmandu) | Tertiary Hospital ANC | Retrospective longitudinal | 4600 | LMP | 15-40 weeks; Westin |
| White (2012)^28^ | Thailand (Maela Refugee Camp, Thai-Burma border; *Karen and Burmese ethnicity*) | Primary Health Clinic/ peripheral facility | Prospective | 2437 (7476) | Ultrasound (8-21 weeks) | $\geq$8 weeks |
| **Latin American Studies** | | | | | | |
| da Cunha (1985)^29^ | Brazil (Ribeirão Preto; *excluded preterm infants*) | Tertiary Hospital ANC | Retrospective | 180 | LMP | 10-44 weeks |
| **Multiregional LMIC Studies** | | | | | | |
| Althabe (2015)^30^ | Argentina (Buenos Aires), DRC (Kinshasa), India (Belgaum), Pakistan (Karachi), Zambia (Lusaka) | Tertiary Hospital | Cross-sectional, targeted sampling for term & preterm distribution | Argentina: 284,  DRC: 671, India: 253, Pakistan: 250, Zambia: 242 | Ultrasound (<20 weeks) | 20-23, 24-36, and >36 weeks; Villar technique |
| **High Income Countries** | | | | | | |
| Baeyertz (1983)^31^ | New Zealand (Wanganui) | Tertiary Hospital | Longitudinal | 87 (109) | Ultrasound (8-14 weeks) | 8-40 weeks |
| Engstrom (1993)^32^ | USA (Chicago, IL) | Tertiary Hospital | Cross sectional | 60 | LMP (Not clearly stated) | 16-42 weeks; three Varley techniques |
| Jelks (2007)^33^ | USA (Torrence, CA) | Tertiary Hospital | Prospective | 103 (206 or 412) depending on the analysis | BOE (LMP & Ultrasound)  (<26 weeks) | 24-40 weeks |
| Jimenez (1983)^34^ | USA (Dallas, TX; *Mexican American women*) | Primary Health Center | Prospective longitudinal | 80 | LMP | 7-42 weeks |
| Ngan (1988)^35^ | Hong Kong | Tertiary Hospital ANC | Prospective | 69 (138) | LMP | 14-42 weeks |
| Papageorghiou^1^ (2016)^36; INTERGROWTH^ | Brazil (Pelotas), China (Beijing), India (Nagpur), Italy (Turin), Kenya (Nairobi), Oman (Muscat), UK (Oxford), USA (Seattle) *Healthy, well-nourished selected population* | Tertiary Hospitals | Prospective longitudinal | 4239 (20566) | BOE (LMP & Ultrasound)  (9-19 weeks) | 14-40 weeks; Villar technique |
| Rogers (1985)^37^ | UK (Birmingham) | Tertiary Hospital | Prospective | 95 (265) | LMP or Ultrasound (<25 weeks) | 17-41 weeks |

| **Excluded studies reporting upon the accuracy of Symphysis Fundal Height to estimate Gestational Age** | | | | | | |
| --- | --- | --- | --- | --- | --- | --- |
| **Author (Year)** | **Location**  Country (district, town) | **Study Setting** | **Study Design** | **Sample Size** | **Key Findings** | **Reason for Exclusion** |
| Basso (2016)^38^ | Brazil (Sao Paulo) | Tertiary hospital | Cross-sectional | 206 | Prediction error for various combinations of anthropometric measures ranged between 13.9 and 14.9 days. Longitudinal fundal height alone predicted gestational age within 14.9 days. Correctly identified 75% of preterm births. | Included only diabetic and hyperglycemic patients |
| Fescina  (1984, 1987)^39,40^ | Uruguay (Montevideo) | Out-patient clinic | Longitudinal | 47 | Sensitivity for SGA = 56%, Specificity = 91%, PPV = 80%, and NPV = 77% | Included only high-risk, diabetic and hypertensive patients, and sample <50. |
| Ogbe (2015)^41^ | Nigeria (Jos) | Maternity unit of the Jos University Teaching Hospital | Cross-sectional prospective | 289 | The mean percentage accuracy for SFH method compared to ultrasound scan dating was 95.8% (p-value = .02). The mean difference comparing the accuracy of SFH and ultrasound for the first measure was -0.028 (SD 1.504, 95% CI (-0.202 – 0.146), p = 0.755), and for the second measure was -0.031 (SD 1.449, 95% CI (-0.199 – 0.137), p = 0.715). | Excluded IUGR and complicated pregnancies |
| Papageorghiou^1^ (2016); ***INTERGROWTH*** | Brazil (Pelotas), China (Beijing), India (Nagpur), Italy (Turin), Kenya (Nairobi), Oman (Muscat), UK (Oxford), USA (Seattle) | Tertiary hospitals, 8 sites | Prospective longitudinal | 4239 | Estimation of gestational age from SFH was expressed by the equation: GA (exact weeks) = 6.585838 − 2.7072585 Å~ (SFH0.5) + 1.295291 Å~ (SFH). Assessment of the goodness of fit showed excellent agreement. Analysis of the duplicate SFH measurements obtained from all women showed the 95% LOA were about 1.5 cm. | Highly selected population, included only healthy pregnancies with strict BMI and height restrictions |
| Pugh (2018)^42^ | USA (NY, DE, MA, CA, NJ, SC, AL, IL, RI) | Tertiary hospitals, 12 sites | Prospective cohort | 2224 | Prediction error for various combinations of anthropometric measures ranged between 13.9 and 14.9 days. Longitudinal fundal height alone predicted gestational age within 14.9 days. Correctly identified 75% of preterm births. | Included only healthy non-obese, low-risk patients |
| Unger (2019)^43^ | Burkina Faso, Ghana, Malawi, Zambia | Hospitals | Secondary analysis of RCT data | 1624 | Correlation coefficient = 0.63, mean difference = 0.40 weeks, and 95% LOA = –4.9 to 5.8 weeks. Sensitivity = 0.80 (95%CI 0.74–0.85), Specificity = 0.74 (95%CI 0.72– 0.76), PPV = 0.35 (95%CI 0.31–0.39) and NPV= 0.96 (95%CI 0.94–0.97). | Included only confirmed *p. falciparum* malaria patients in 2^nd^ or 3^rd^ trimester |

^1^Papageoghiou et al. (2016) is included in Appendix S1: Table 4. Inter-rater and Intra-rater Reliability Assessments but was excluded from Table 2. Studies reporting upon the accuracy of SFH to estimate GA

Abbreviations: ANC = antenatal care, BOE = best obstetric estimate, CI = confidence interval, GA = gestational age, LMP = last menstrual period, LOA = limits of agreement, NPV = negative predictive value, PPV = positive predictive value, SFH = symphysis-fundal height, SGA = small for gestational age.

**S2 Table. Methods for Converting or Calculating Gestational Age from Symphysis Fundal Height**

| **Author and Year** | **Country** | **Study Sampling** | **Sample Size** | **Gold Standard (U, BOE, LMP)** | **Mean or Median** | **Equation/Conversion Method** |
| --- | --- | --- | --- | --- | --- | --- |
| **Clinical Rules/Standards** | | | | | | |
| Douglas (2006)^44^  1-1 conversion | UK | NS | NS | NS | NS | SFH=GA+/-3ccm |
| Fournié (1987, 2004, 2007)^45^  Rule of 4 | France, used in Rwanda | NS | NS | NS | NS | Also used in Rwanda, and referenced in the French Association of Gynecologists and Obstetricians syllabus for residents, and in an online calculator (<http://medicalcul.free.fr/agegest_hu.html>);   - 16cm=20wks, increases 1cm per week between 20 and 32 weeks, and 0.5cm per week until birth. Therefore, SFH=GA-4 - OR; number of months of pregnancy x 4 for months 4 to 7; month 8=30cm; month 9=32-34cm   OR; between 16 and 32 weeks GA, normal uterine height ranges from (number of completed GA weeks – 4) to (number of completed GA weeks +1); i.e., at 30 weeks SFH ranges from 26 to 31 cm |
| McDonald (1906)^46^ 1-1 up to 32 wks | USA | NS | NS | NS | NS | GA=cm up to 32 weeks GA, then SFH increases 1cm every 2 weeks |
| **Statistical Methods** | | | | | | |
| Deeluea (2013) | Thailand | Retrospective time-series | 1038 (7523) | LMP or U (648 LMP; 390 U) | Median | SFH=-19.7882+2.438157(GA)-0.0262178(GA*GA) |
| Lee (2020) | Bangladesh | Prospective, consecutive, community-based recruitment | 1146 (3480) | U | Mean | GA=23.34201(lnSFH)-45.0998 |
| Mador (2010) | Nigeria | Cross-sectional systematic sampling | 405 (405) | U | Mean | SFH=(0.0024*(GA*GA)+1.1255*(GA)-1.8334) |
| Ogunranti (1990) | Nigeria | Prospective | 581 | LMP & Date of Birth | 50^th^ | GA=12.4+0.7(SFH) |
| International Standard (INTERGROWTH)  Papageorghiou (2016) | Brazil, China, India, Italy, Kenya, Oman, UK, USA (extremely healthy women and infants) | Prospective longitudinal, consecutive | 4239 (20566) | BOE (U & LMP) | Median | SFH=5.133374 + 0.1058353119 × (GA*GA) – 0.0231295 × (GA*GA) × ln (GA); SD (SFH) = 0.9922667 + 0.0258087 * GA  Inverse Equation: GA(exact weeks)=6.585838-2.7072585*(SFH^0.5)+1.295291*SFH |
| Sogbanmu (1980) | Nigeria (Xhosa) | Random | 159 | LMP (Not clearly stated) | Mean | GA=0.87(SFH)±5.18 |
| van Bogaert (1999) | South Africa (Xhosa) | Cross-sectional | 800 | U | Median | SFH=2.45+0.87(GA); (SEE=±2.62) |
| **Other Methods** | | | | | | |
| Althabe (2015) | DRC, India, Pakistan (and tested in Argentina, India, Pakistan, Zambia) | Prospective | NS | U | NA | Color-coded tape: yellow zone (GA <24 weeks), red zone (GA 24-36 weeks), green zone (GA >36 weeks); Red zone is the "GA period in which antenatal corticosteroids should be administered if a woman showed signs of labor or pregnancy complications." |
| White (2012) | Thailand (Thai-Burma border; Karen women) | Prospective | 2437 (7476) | U | Mean | Online Shoklo symphysis fundal height calculator (<https://www.tropmedres.ac/units/moru-bangkok/mathematical-and-economic-modelling/gestational-age>) |
| Sample size was number of pregnant women in the study, and the number of measurements in the study is given in parentheses. Abbreviations: SFH= symphysis fundal height (in cm), GA= gestational age (in weeks), U= ultrasound, LMP= last menstrual period, BOE= best obstetric estimate, DRC=Democratic Republic of Congo, NS= not stated. Sample size: number of women in the study, and number of measurements in parentheses | | | | | | |

**S3 Table. Population-based Reference Data of SFH Measurements (cm) by LMP/BOE estimated Gestational Age (weeks) in Low-Middle Income Countries, by Region**
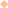

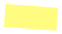

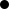


| **Author & Year** | **Country & Study Setting** | **Sample size** | **Mean/ Median** | **20** | **22** | **24** | **26** | **28** | **30** | **32** | **33** | **34** | **35** | **36** | **37** | **38** | **39** | **40** | **41** |
| --- | --- | --- | --- | --- | --- | --- | --- | --- | --- | --- | --- | --- | --- | --- | --- | --- | --- | --- | --- |
| **African Studies** | | | | | | | | | | | | | | | | | | | |
| Ebite (2009) | Uromi, Nigeria | 202 | Mean† | 20.4 (2.0) |  | 24.4 (2.0) |  | 28.2 (2.0) |  | 32.1 (2.0) |  |  |  | 35.4 (2.0) |  |  |  | 36.6 (2.0) |  |
| Ogunranti (1990) | Port Harcourt, Nigeria | 581 | 50^th^♦◊ |  |  |  |  |  | 26.5 |  |  |  | 30.0 | 32.0 | 33.7 | 36.8 | 39.0 |  |  |
| Sogbanmu (1980) | Ondo, Nigeria | 159 | Mean◊ |  |  |  | 24.0 |  |  |  |  | 33.0 |  |  |  |  |  |  |  |
| **Asian Studies** | | | | | | | | | | | | | | | | | | | |
| Deeluea (2013) | Bangkok, Chiang Mai, & Phayao, Thailand | 1038 (7523) | Mean†♦ | 19.1 (1.9) | 21.5 (2.1) | 23.7 (1.7) | 25.3 (1.7) | 27.7 (1.8) | 29.7 (1.7) | 31.5 (1.6) | 32.2 (1.7) | 33.1 (1.6) | 33.8 (1.7) | 34.4 (1.7) | 34.9 (1.9) | 35.0 (2.0) | 35.2 (2.3) | 35.4 (2.4) |  |
|  |  |  | 50^th^♦ | 18.7 | 21.3 | 23.7 | 25.9 | 28.0 | 29.8 | 31.4 | 32.0 | 32.8 | 33.4 | 34.0 | 34.5 | 35.0 | 35.4 | 35.8 |  |
| Agarwal (2002) | Varanasi, India | 3700 (11100) | Mean† (Curve) | 14.1 (2.5) | 16.1 (2.8) | 20.2 (0.9) | 23.8 (1.3) | 26.6 (1.0) | 29.2 (1.1) | 30.1 (1.3) | 30.7 (1.1) | 31.4 (0.9) | 31.7 (1.4) | 31.3 (1.2) | 31.7 (1.3) | 31.7 (1.5) |  |  |  |
| Fikree (1988) | Karachi, Pakistan | 163 | Mean† | 16.0 (3.7)  *<5 mo* |  | 24.3 (3.4)  *6-7 mo* | | |  | 32.1 (5.2)  *8+ mo* | | | | | | | | | |
| Ghate (1995) | Mangalwar Peth & Sadashev Peth, India | 281 (281) | Mean† |  |  |  | 25.4 (7.8) | 26.9 (4.4) | 28.6 (6.7) | 30.7 (5.1) |  | 31.9 (5.6) |  | 33.2 (3.7) |  | 34.2 (5.7) |  | 35.1 (8.6) |  |
| Indira (1990) | Pondicherry, India | 109 (706) | Mean† (Curve) | 15.4 (2.0) | 18.6 (2.1) | 21.4 (1.9) | 24.1 (2.1) | 26.7 (2.0) | 28.3 (2.3) | 30.0 (2.5) | 31.0 (2.2) | 31.7 (2.4) | 32.4 (2.3) | 33.0 (2.4) | 33.5 (2.2) | 33.8 (2.3) | 34.0 (2.1) | 34.0 (2.2) | 34.1 (1.5) |
| Ulstein (1988) | Kathmandu, Nepal | (4600) | Mean (Curve) | 16.7 | 18.7 | 21.0 | 23.0 | 25.1 | 27.1 | 28.5 | 29.0 | 29.6 | 29.8 | 30.2 | 30.4 | 30.6 | 30.9 | 31.0 |  |
| Mathai (1987) | Vellore, India | 250 (584) | Mean (Curve) † |  | 20.0 (3.4) | 21.5 (3.5) | 22.9 (3.2) | 24.4 (2.9) | 26.1 (2.6) | 27.9 (2.8) | 28.9 (2.9) | 29.8 (2.8) | 30.8 (2.9) | 31.5 (2.8) | 32.3 (2.8) | 32.9 (2.8) | 33.3 (2.9) | 33.7 (3.0) |  |
| **Latin American Studies** | | | | | | | | | | | | | | | | | | | |
| da Cunha (1985) | Sao Paulo, Brazil | (180) | Median (Curve) | 20.5 | 20.9 | 23.5 | 24.7 | 27.3 | 28.5 | 30.4 | 32.2 | 31.4 | 32.4 | 33.3 | 32.4 | 34.5 | 34.9 | 35.3 | 35.3 |
| An empty cell indicates that the data was not available for that paper. Sample size was number of pregnant women in the study, and number of measurements is in parentheses. Abbreviations: SFH= symphysis fundal height, GA= gestational age, U= ultrasound, BOE= best obstetric estimate, LMP= last menstrual period, DRC=Democratic Republic of Congo.  * Indicates inclusion in weighted population-based reference curve created in the current study (with shading in Mean/Median column; only Means from studies with ultrasound as the gold standard reference were included in the weighted population-based reference curve for this study); † Indicates paper also has standard deviations for each gestational age week listed; ‡ Indicates paper also has standard error for each gestational age week listed;∇ Indicates study excluded preterm infants.  ♦ Indicates paper also has sample size (number of women) for each gestational age week listed;  ◊ Indicates population-based reference data study was presented in an inverted table (listed average weeks of gestational age for each whole SFH cm measurement).  ** Pelotas, Brazil; Beijing, China; Nagpur, India; Turin, Italy; Nairobi, Kenya; Muscat, Oman; Oxford, UK; Seattle, USA; institutions providing obstetric care with no or low levels of major, known, non-microbiological contamination. | | | | | | | | | | | | | | | | | | | |

**S4 Table. LMP-based studies reporting upon the accuracy of Symphysis Fundal Height to estimate Gestational Age**

| **Author** | **Year** | **Study Setting** (NICU/clinic/hospital/ community, district/city, country) | **Sample Size** | **SFH Measurement/**  **conversion to GA** | **GA estimated by SFH versus reference GA method** | | | | | | | **Validity to identify preterm GA**  **(<37 weeks unless otherwise noted)** | | | | | | |
| --- | --- | --- | --- | --- | --- | --- | --- | --- | --- | --- | --- | --- | --- | --- | --- | --- | --- | --- |
|  |  |  |  |  | **Correlation** (R) with reference GA | Mean difference/bias (days) [SFH - reference GA) | SD of the mean GA difference (days) | Bland Altman 95% LOA (LL, UL) [days] | 95% CI prediction error | % within 7 days | % within 14 days |  |  |  |  |  |  |  |
|  |  |  |  |  |  |  |  |  |  |  |  | Sensitivity (%) | Specificity (%) | | PPV  (%) | | NPV  (%) | |
| **LMP as Reference** | | | | | | | | | | | | | | | | | | |
| **High Income Countries** | | | | | | | | | | | | | | | | | | |
| Jimenez | 1983 | Primary health center, Dallas, TX, USA | 80 | Polynomial regression model | – | 0.6 | 8.4 | – | – | – | – | – | | – | | – | | – |
| Baeyertz | 1983 | Tertiary hosp, N Zealand | 127 | NS | – | – | 10 | – | – | 60 | 79 | – | | – | | – | | – |
| Da Cunha | 1985 | Tertiary hospital, Brazil | 180 | NS | 0.93 | – | – | – |  | – | – | – | | – | | – | | – |
| **Low/Middle Income Countries (LMIC)** | | | | | | | | | | | | | | | | | | |
| Adewale | 2011 | Tertiary hospital, Nigeria | 390 | NS | – | – | – | – | – | – | 80 | – | | – | | – | | – |
| Amagloh | 2009 | Tertiary Hospital, Ghana | 100 | NS | 0.29 | – | – | – | – | – | – | – | | – | | – | | – |
| Rada | 2018 | Benin, Gabon, Mozambique, Tanzania | 3038 | NS | – | 2.8 | 32.4 | (-60.6, 66.2) | – | – | – | – | | – | | – | | – |
| Sogbanmu | 1980 | Tertiary hospital, Ondo State, Nigeria | 159 | NS | 0.90 | – | – | – | – | – | – | – | | – | | – | | – |
| (–) indicates that the data was not available for that paper  *Numbers were calculated by authors of this paper  ^1^Concordance defined by American College of Obstetricians and Gynecologists: <7 days between 14-15 weeks, <10 days between 16-21 weeks, and <14 days between 22-27 weeks  Abbreviations: SFH= symphysis fundal height, NS= not stated, GA= gestational age, AGA= appropriate-size-for-gestational age, SD= standard deviation, LOA= limits of agreement, LL=lower limit, UL=upper limit, CI= confidence interval, PPV= positive predictive value, NPV=negative predictive value, BOE= best obstetric estimate (LMP confirmed by ultrasound or clinical evaluation), LMP= last menstrual period | | | | | | | | | | | | | | | | | | |

**S5 Table. Inter-rater and Intra-rater Reliability Assessments**

| **Author** | **Study Setting** | **Sample Size** | **SFH Measurements Technique and Timing** | **Gold Standard (U, BOE, LMP)** | **Type of Health Worker and Description of repeat measures** | **Bland Altman agreement, Correlation coefficient for Inter-rater or Intra-rater, or Other** |
| --- | --- | --- | --- | --- | --- | --- |
| **Inter-rater** | | | | | | |
| Althabe (2015) | Kinshasa, DRC; Karnataka, India; Karachi, Pakistan | NS | Fundus defined by hand. Supine position. Color-coded tape, from upper edge of the pubic bone to center-point of uterine fundus (30-50 women for each GA week) | U | NS: 2 operators; | 95% within 2-3cm (Bland Altman Plots for interrater reliability available, but no related numbers stated in the paper) |
| Engstrom (1993) | Chicago, IL, USA | 60 for tape measurements, 59 for caliper measurements | Measurer blinded to tape/caliper, own measurements, and other readers’ measurements. Supine position, measurement from uppermost border of SP to uppermost border of uterine fundus in midline. Tape “over the curve” method: tape in contact with skin for the entire curve. Tape “not over the curve” method: tape in contact with skin until the curve of the uterine fundus was reached; at that point, the tape was extended in a straight line from the fundus to the point perpendicular to the uppermost border of the uterine fundus. Caliper method: the distance between the innermost points of the branches of the caliper was measured for landmarks as described above. (16-42 weeks) | LMP (Not clearly stated) | 1 obstetrician, 3 nurse midwives. 2 measurements per reader. | *Tape “over the curve” method:* Mean difference (MD) 2.06 cm (range MD 1.40 to 2.56 cm, range SD of MD 1.14 to 3.40 cm), 20-38.9% within 1 cm; 50-72% within 2 cm  *Tape “not over the curve” method:* MD 2.50 cm (range MD 1.36 to 3.60 cm, range SD of MD 1.58 to 3.37 cm); 9.1-55.6% within 1 cm; 13.6-72.2% within 2 cm  *Caliper method:* MD 2.14 cm (range MD 1.37 to 3.05 cm, range SD of MD 1.66 to 3.11 cm); 15.0-55.0% within 1 cm; 25.0-77.8% within 2 cm |
| Fikree (1988) | Karachi, Pakistan | 163 (326) | NS | LMP | Faculty members (5) vs. nursing students (20) | Inter-rater correlation coefficient: 0.981, p<0.0001 |
| Jelks (2007) | Torrence, CA, USA | 103 (206-412) | NS (24-40 wks) | BOE (U & LMP) | Clinicians (Medical students; Obst & Gyn residents, nurse midwives, faculty; maternal-fetal medicine fellows): Each observer obtained one measurement. Study made comparisons between juniors, seniors, and with same level of experience; with blank or marked tapes; Juniors had ≤2 years residency or were medical students, Seniors had 3-4 years residencies, or were faculty/fellow, nurse practitioner/midwives | Kappa inter-rater: ranges between 0.29 and 1.60 |
| Lee (2020) | Sylhet, Bangladesh | 131 (262) | Emptied bladder, supine position; palpated superior rim of pubic bone, then palpated uppermost point of uterine fundus, marked points with a pen, measured vertical axis crossing umbilicus. Repeated 3 times. (20-36 wks) | U | Physicians vs CHWs: Compared physician measurements to those of CHWs in a random sample of 131 women in the study, each taking 3 SFH measurements | Mean difference: 0.88cm (95% LOA ±3.65); 70% within 2 cm |
| Ngan (1988) | Hong Kong | 69 (138) | Fundus defined by the hand. Measured from upper border of the pubic symphysis and gently stretched over the midline of the abdomen. (32-38 wks) | “Certain” LMP | Physicians: 2 observers (the authors) | Range of differences in SFH measurements between the observers was 0 to 2.5 cm. The absolute percentage difference ranged from 0% to 9.5%. The mean percentage difference was 2.7 +/- 2.5% SD |
| Rogers (1985) | Birmingham, UK | 95 (265) | Uterus defined by palpation. Measured from xiphisternum to upper border of symphysis pubis, woman in supine position. Mostly midline measurements. Oblique if highest point of uterus was to either side of midline. (17-41 wks) | LMP or U | Physicians, auxiliary nurses, staff midwife: Compared physician measurements to those of an auxiliary nurse or staff midwife. 67% of measurements by auxiliary nurses. | Mean difference: 0.66cm (SD ±1 cm); precision (95% CI) was therefore ±2 cm |
| Shamawarna (2012) | Galle, Sri Lanka | 400 (800) | Measured from the top of the fundus to the top of symphysis pubis along longitudinal uterine axis (20-41 wks) | U | First author or Senior house officer vs. other house officers: 2 observers | Reliability Coefficient Alpha=0.9774, p<0.0001 (no significant inter-observer variation; highly correlated) |
| **Intra-rater** | | | | | | |
| Engstrom (1993) | Chicago, IL, USA | 60 for tape measurements, 59 for caliper measurements | Measurer blinded to tape/caliper, own measurements, and other readers’ measurements. Supine position, measurement from uppermost border of SP to uppermost border of uterine fundus in midline. Tape “over the curve” method: tape in contact with skin for the entire curve. Tape “not over the curve” method: tape in contact with skin until the curve of the uterine fundus was reached; at that point, the tape was extended in a straight line from the fundus to the point perpendicular to the uppermost border of the uterine fundus. Caliper method: the distance between the innermost points of the branches of the caliper was measured for landmarks as described above. (16-42 weeks) | LMP (Not clearly stated) | 1 obstetrician, 3 nurse midwives. 2 measurements per reader. | *Tape “over the curve” method*: MD 1.13 cm (Range of MD is 0.82 to 1.50 cm; Range of SD for MD is 1.07 to 1.89 cm)  *Tape “not over the curve” method*: MD 1.21 cm (Range of MD is 0.84 to 1.74 cm; Range of SD for MD is 0.93 to 1.75 cm)  *Caliper method*: MD 0.92 cm (Range of MD is 0.68 to 1.39 cm; Range of SD for MD is 0.84 to 1.89 cm) |
| Limpanyalert & Manotaya (2001) | Bangkok, Thailand | 199 (879) | Westin. Supine position (16-40 wks) | BOE (LMP & Ul) | Physician: All measurements by 1 author | ICC=0.99 |
| Papageorghiou (2016)  (INTERGROWTH) | Brazil, China, India, Italy, Kenya, Oman, UK, USA (tertiary hospitals) | 4239 (20566) | From upper border of symphysis pubis, straight line over uterus to fundus. Supine position. (14wks-birth) | BOE | Research staff: NS | Duplicate measures had 95% LOA of 1.5cm. Inter-rater agreement: 0.07cm (95% LOA: 0.66cm, 0.81cm) |

**S1 Fig. QUADAS-2 summary for studies reporting agreement between SFH and gold standard GA**

|  | **Risk of Bias from Patient Selection** | **Risk of Bias from Test Method** | **Risk of Bias from Reference Standard** | **Risk of Bias from Flow and Timing** |
| --- | --- | --- | --- | --- |
| **Low** | 9 | 2 | 10 | 14 |
| **Unclear** | 3 | 6 | 2 | 2 |
| **High** | 5 | 9 | 5 | 1 |

*Individual study assessments can be shared upon request

**S1 Appendix References**

1. Adewale FB, Ijaiya MA. Symphysio-fundal height measurement as a means of gestational age assessment in the second half of pregnancy at the University of Ilorin Teaching Hospital, Nigeria. Bangladesh Journal of Obstetrics and Gynecology. 2011;26(1):3-9.

2. Amagloh FK, Williams AA, Angbing I. Evaluation of some maternal and socio-economic factors associated with low birthweight among women in the Upper East Region, Ghana. African Journal of Food Agriculture Nutrition and Development. 2009 2009 Oct;9(7):1498-510.

3. Challis K, Osman NB, Nyström L, Gunnar N, Bergstrm S. Symphysis-fundal height growth chart of an obstetric cohort of 817 Mozambican women with ultrasound-dated singleton pregnancies. Tropical Medicine and International Health. 2002;7(8):678-84.

4. Ebite LE, Ebeigbe PN, Igbigbi P, Akpuaka FC. Symphysiofundal height growth curve and growth velocity in pregnant women in a Nigerian community. Journal of Obstetrics and Gynaecology. 2009;29(7):605-8.

5. Kiserud T. Fundal height growth in rural Africa. Acta Obstet Gynecol Scand. 1986;65(7):713-5.

6. Mador E, Pam S, Pam I, Mutihir J, Adoga G, Ogunranti J. Symphysio-Fundal Height Nomogram In Ultrasound Dated Pregnancies. Asian Journal of Medical Sciences. 2011 01/20;1.

7. Malaba TR, Newell ML, Madlala H, Perez A, Gray C, Myer L. Methods of gestational age assessment influence the observed association between antiretroviral therapy exposure, preterm delivery, and small-for-gestational age infants: a prospective study in Cape Town, South Africa. Ann Epidemiol. 2018 12;28(12):893-900.

8. Ogunranti JO. Fundal height in normal pregnant Nigerian women: anthropometric gravidogram. Int J Gynaecol Obstet. 1990 Dec;33(4):299-305.

9. Rada S, Gamper J, González R, Mombo-Ngoma G, Ouédraogo S, Kakolwa MA, et al. Concordance of three alternative gestational age assessments for pregnant women from four African countries: A secondary analysis of the MIPPAD trial. PloS one. 2018;13(8):e0199243.

10. Sogbanmu MO. Simple method of assessment of fetal maturity in a rural community in Nigeria. East Afr Med J. 1980 May;57(5):346-9.

11. van Bogaert LJ. Customised gravidogram and fetal growth chart in a South African population. Int J Gynaecol Obstet. 1999 Aug;66(2):129-36.

12. van Rensburg YJ, Botha D, Nel R, Fichardt A. A comparison of skilled methods to determine gestational age in pregnancy. Curationis. 2003;26(1):22-6.

13. Agarwal S, Agarwal A, Bansal AK, Agarwal DK, Agarwal KN. Birth weight patterns in rural undernourished pregnant women. Indian pediatrics. 2002 Mar;39(3):244-53.

14. Deeluea J, Sirichotiyakul S, Weerakiet S, Arora R, Patumanond J. Fundal height growth curve for underweight and overweight and obese pregnant women in Thai population. ISRN Obstetrics and Gynecology. 2013;2013.

15. Fikree F, De Onis M, Marshall P, Badrudding S, Bryant J, Berendes H, et al. A rapid nutritional evaluation of pregnant women in urban areas in developing countries: [Unpublished] [1988]. 1988.

16. Ghate M, Pratinidhi A, Gupte A. Risk prediction charts for low birth weight. Indian pediatrics. 1996 Jan;33(1):15-8.

17. Indira R, Oumachigui A, Narayan KA, Rajaram P, Ramalingam G. Symphysis-fundal height measurement - A reliable parameter for assessment of fetal growth. International Journal of Gynecology and Obstetrics. 1990;33(1):1-5.

18. Jehan I, Zaidi S, Rizvi S, Mobeen N, McClure EM, Munoz B, et al. Dating gestational age by last menstrual period, symphysis-fundal height, and ultrasound in urban Pakistan. Int J Gynaecol Obstet. 2010 Sep;110(3):231-4.

19. Karl S, Suen C, Unger HW, Ome-Kaius M, Mola G, White L, et al. Preterm or Not - An Evaluation of Estimates of Gestational Age in a Cohort of Women from Rural Papua New Guinea. Plos One. 2015 May;10(5).

20. Lee ACC, Whelan R, Bably NN, Schaeffer LE, Rahman S, Ahmed S, et al. Prediction of gestational age with symphysis-fundal height and estimated uterine volume in a pregnancy cohort in Sylhet, Bangladesh. BMJ open. 2020 03;10(3):e034942.

21. Limpanyalert P, Manotaya S. Standard curve of symphysial-fundal height measurement and pregnancy characteristics in pregnant women at King Chulalongkorn Memorial hospital. Thai Journal of Obstetrics and Gynaecology. 2001;13(4):197-206.

22. Mathai M, Jairaj P, Muthurathnam S. Screening for light-for-gestational age infants: a comparison of three simple measurements. Br J Obstet Gynaecol. 1987 Mar;94(3):217-21.

23. Moore KA, Simpson JA, Thomas KH, Rijken MJ, White LJ, Dwell SL, et al. Estimating Gestational Age in Late Presenters to Antenatal Care in a Resource-Limited Setting on the Thai-Myanmar Border. PLoS One. 2015;10(6):e0131025.

24. Rao T, Doppa G, Gowder R. Case Study - Is There a Need to Customize Gravidogram Based on Ethnic Population? [Poster]. RCOG World Congress; 2014. [Accessed May 26,2022]: https://epostersonline.com/rcog2014/node/2588

25. Shamawarna KH, Goonewardene IM, Perera YA. Customised symphysio fundal height charts. The Ceylon medical journal. 2012 Dec;57(4):159-65.

26. Shrestha R, Andrews-Trevino JY, Acharya S, Lamichhane A, Pokharel A, Davis D, et al. Estimating gestational age using last menstrual period (LMP) and symphysis fundal height (SFH) measures rather than ultrasound examination: A test of methods for use in low income settings. Annals of Nutrition and Metabolism. 2017;71:762-3.

27. Ulstein M, Rana G, Yangzom K, Gurung R, Karki A, Gurung G, et al. Some fetal and pregnancy parameters in Nepal. Acta Obstet Gynecol Scand. 1988;67(1):47-52.

28. White LJ, Lee, S.J, Stepniewska, K., Simpson, J.A., Dwell, S.L.M., Arunjerdja, R., Singhasivanon, P., White, N.J., Nosten, F., McGready, R. Estimation of gestational age from fundal height: a solution for resource-poor settings. Journal of the Royal Society Interface. 2012;9:503-5010.

29. da Cunha SP, Ribeiro JU, Berezowski AT, Duarte G. Evolution of uterine height and abdominal circumference in normal pregnant women. Revista paulista de medicina. 1985;103(5):231-4.

30. Althabe F, Berrueta M, Hemingway-Foday J, Mazzoni A, Bonorino CA, Gowdak A, et al. A color-coded tape for uterine height measurement: A tool to identify preterm pregnancies in low resource settings. PLoS ONE. 2015;10(3).

31. Baeyertz JD. Assessment of gestational maturity using a new fundal height measuring tape. The New Zealand medical journal. 1983 Dec 28;96(746):1059-60.

32. Engstrom JL, McFarlin BL, Sittler CP. Fundal height measurement. Part 2--Intra- and interexaminer reliability of three measurement techniques. J Nurse Midwifery. 1993 1993 Jan-Feb;38(1):17-22.

33. Jelks A, Cifuentes, R., Ross, M.G. Clinician bias in fundal height measurement. Obstetrics & Gynecology. 2007;110(4):892-9.

34. Jimenez JM, Tyson JE, Reisch JS. Clinical measures of gestational age in normal pregnancies. Obstet Gynecol. 1983 Apr;61(4):438-43.

35. Ngan HYS, Woo JSK, Fung KP, Au KKL. A symphysis-fundal height nomogram for Hong Kong Chinese. Journal of the Hong Kong Medical Association. 1988;40(1):55-7.

36. Papageorghiou AT, Ohuma EO, Gravett MG, Hirst J, da Silveira MF, Lambert A, et al. International standards for symphysis-fundal height based on serial measurements from the Fetal Growth Longitudinal Study of the INTERGROWTH-21st Project: prospective cohort study in eight countries. BMJ (Clinical research ed). 2016;355.

37. Rogers MS, Needham PG. Evaluation of fundal height measurement in antenatal care. Aust N Z J Obstet Gynaecol. 1985 May;25(2):87-90.

38. Basso NAD, Morceli G, Costa R, Dias A, Rudge MVC, Calderon IMP. Validation of a symphysis-fundal height chart developed for pregnancy complicated by diabetes and hyperglycemia: an observational study. Reproductive health. 2016 Aug;13.

[39. Fescina RH, Quevedo C, Martell M, Nieto F, Schwarcz R. Uterine height as a method of predicting fetal growth. Boletín de la Oficina Sanitaria Panamericana Pan American Sanitary Bureau. 1984;96(5):377–86.](https://sciwheel.com/work/bibliography/11457532)

40. Fescina RH, Martell M, Martinez G. Small for dates: Evaluation of different diagnostic methods. Acta Obstetricia et Gynecologica Scandinavica. 1987;66(3):221-6.

41. Ogbe AE, Ekwempu CC, Musa J, Anzaku AS. A Comparison of the Accuracy of the Use of Last Menstrual Period and Symphysio-Fundal Height for Gestational Age Determination among Nigerian Women. 2015 2015/12/03.

42. Pugh SJ, Ortega‐Villa AM, Grobman W, Newman RB, Owen J, Wing DA, et al. Estimating gestational age at birth from fundal height and additional anthropometrics: a prospective cohort study. BJOG: An International Journal of Obstetrics & Gynaecology. 2018;125(11):1397-404.

43. Unger H, Thriemer K, Ley B, Tinto H, Traoré M, Valea I, et al. The assessment of gestational age: a comparison of different methods from a malaria pregnancy cohort in sub-Saharan Africa. BMC pregnancy and childbirth. 2019;19(1):1-9.

44. Douglas G, Nicol F, Robertson C. Macleod's Clinical Examination: Elsevier Health Sciences; 2006.

45. Fournié A, Lefebvre-Lacoeuille C, Cotici V, Harif M, Descamps P. The fundal height measurements in single pregnancies and the detection of fetal growth retardation. J Gynecol Obstet Biol Reprod (Paris). 2007 Nov;36(7):625-30.

46. McDonald E. Mensuration of the Child in the Uterus with New Methods. Journal of the American Medical Association. 1906;47(24):1979-83.
