# Supplementary material for: Measurement of symphysis fundal height for gestational age estimation in low-to-middle-income countries: A systematic review and meta-analysis
Source: PLoS One. 2022 Aug 25;17(8):e0272718. doi: 10.1371/journal.pone.0272718 (PMC9409500; doi:10.1371/journal.pone.0272718)
Supplement: Attachment — Submitted filename: SFH Plos Med Review Response.pdf [file pone.0272718.s003.pdf]

Re: Measurement of symphysis fundal height for gestational age estimation in Low-to-Middle-Income Countries: A systematic review and meta-analysis

October 8, 2021,

Dear Plos One Editor,

We appreciate your consideration of our manuscript at Plos One. Our manuscript was favorably reviewed at Plos Medicine by 4 reviewers. We appreciate the positive feedback and opportunity to respond point by point below.

REVIEWER #1: The paper is excellently done and provides a thorough evaluation of fundal height measurements for gestational age determination. I have no criticisms.

Author Response: We thank the reviewer for the positive comments.

REVIEWER #2: This systematic review summarized the relationship between SFH and GA across different regions and assessed the accuracy of estimating GA using SFH in general obstetric populations. I think this is an important topic and the results are informative to clinical practice in the LMICs.

Overall, this is a rigorously designed and conducted SR study. The Authors clearly describe the data seeking and data extraction process and the analyses are rigorous, comprehensive, and easy to understand.

Below are specific comments:

Line 96-98: Is there any evidence about the statement that the distribution is symmetric. May need to provide a few citations.

Author Response: We assessed for symmetry around the median value by evaluating the magnitudes of the surrounding interquartile ranges (25-50% compared to the 50-75%) to assess whether they were equal [Rosner B. Fundamentals of Biostatistics, 8th edition, Chapter 2]. For studies with similar magnitude IQRs, the distribution was considered symmetric, and the median values were used to approximate the mean. Data that was not symmetric was not included in this analysis. We have added this detail to the manuscript (Lines 125-127) as well as citations [Rosner B. Fundamentals of Biostatistics, 8th edition, Chapter 2].

Line 98-101: a) I figured out what "average study" means later from the equation, but I think it can be very helpful to explain that concept here. b) I am wondering what are the benefits of using fixed effects rather than random effects model here? For random effects, we assume the true effect follows a distribution, which can account for more heterogeneity across studies. And

to assess the accuracy of using SFH to estimate GA, one can analyze the between study heterogeneity.

As some additional thoughts, I think this meta analysis can be perfectly done using the MR-BRT method newly developed by IHME (Zheng P, Aravkin A, Barber R, Sorensen R, Murray C. Trimmed Constrained Mixed Effects Models: Formulations and Algorithms. bioRxiv. 2020 Jan 29;2020.01.28.923599.) This is a more flexible random effect model which can include both study-specific and estimate-specific covariates to adjust for biases. It can also apply a range of regularizations to the estimates and can detect and trimmed outliers, which makes it both flexible and robust to outliers. To assess the accuracy, one can model the differences between SFH estimated GA and reference GA using MR-BRT, adjusting all bias covariates, examine the between study heterogeneity and estimate the summarized confidence interval of the differences. In the end, you will have an overall estimate of the difference (adjusting all bias covariates) as well as the uncertainty of the difference. Basing on the mean and uncertainty of the difference, you can draw conclusion on whether the existing evidence warrant the use of SFH or not.

Author Response:

a) We have moved the explanation of the "average study" to earlier in the paragraph (Line 105-108) with additional explanation. We also reference the equation within the text citation to improve clarity, as well as include the reference to Dr. Rosner's original manuscript using this method.

   Line 105-108: "To account for individual study effects, we used our previously described methods[17] to treat the study as a fixed effect and adjusted each mean SFH for the study in order to estimate what the SFH would have been if obtained from an "average study" with "average" study effects (see equation below)."

---

b) We appreciate this suggestion and considered a random effects model. However, we elected to use a more conservative approach with fixed effects for several reasons. The random effects model would require the assumption that the random effect is normally distributed. Given the small number of studies, this was difficult to justify. It is recommended that fixed effects models be used when 5 or fewer studies contribute to a meta-analysis (1). In our pooled analysis there were 4 pooled for Asia, and 5 for Africa, thus we used the fixed effects model. We have added this justification, along with relevant citations, to the methods section of the paper.

Line 117-119: Please describe how the standard error is calculated. Did you use the delta method?

Author Response: The standard error of the logit(proportion) was calculated using the delta method, as per the below equation [Rosner B. Fundamentals of Biostatistics, 8th edition, Chapter 13, page 638]. This equation has been added to the methods in line 147.

se_logit=se_proportion/(proportion(1-proportion))*

Reviewer #3: Accurate gestational age estimation is an important area of current and future research and therefore this systematic review is timely and welcome addition to the body of evidence.

It is a well conducted, comprehensive review and below are some additional comments:

1) The authors highlight the distinction between using SFH for GA estimation (focus of the review) versus monitoring fetal growth. In the included studies LMP is used as part of the reference standard to compare to SFH. One could finish reading the review and think that health workers are using SFH only, with no LMP at all, to estimate GA, which is not the case in clinical practice. Therefore, it would be critical to be clear about the interpretation in the discussion.

Author response: This is a very important point. In this study we only included comparisons of SFH alone to a comparison reference and did not include studies or comparisons of studies that used a combination of SFH and LMP data to estimate GA. We expect that may improve the estimation of gestational age. We have included this limitation in the discussion (Lines 331-333) and also encourage practitioners to consider LMP and other maternal factors in estimating GA in settings where SFH will remain in use and where ultrasound is not routinely available.

2) For standards, the authors use INTERGROWTH-21st, and not WHO Growth Charts published in PLoS Medicine. https://secure-web.cisco.com/1TB6Pl9AHFlnRw0y7DLCIlR2am2uCkHYhqi3k-5eKU7djRIOfKQ7DU4LGqZ3LJY71a4J66FRncFBBqATvGbEp7iOVEZ1ENO8B6inujw-KoFfnilSUjazW4EHcTJovAHrN6vlDYKWIrTZfHizPEOJrkZ9iAY6ota20TsredNUQq0Nfg_Blvk2Q-7ouBzc_KJbP9JIww41uvG6AGOJobXKkVcsrsTqfKBaHkXyQU9gloNjSGzX1DumZylSvfRows8LY/https%3A%2F%2Fjournals.plos.org%2Fplosmedicine%2Farticle%3Fid%3D10.1371%2Fjournal.pmed.1002220. It would be good for transparency to include some kind of justification and also include in the discussion if using WHO growth charts would have made a difference in some of the results being reported and/or interpreted given the reported differences between the two.

Author response: We thank the reviewer for the suggestion. However, the WHO growth charts do not report SFH standard growth charts, only ultrasound-based fetal growth parameters which were outside the scope of our systematic review. To our knowledge, INTERGROWTH-21st is the only source of global standards for SFH in the setting of optimal pregnancy health and fetal growth.

3) Helpful to mention some of the relevant WHO ANC recommendations on early ultrasound or SFH for fetal assessment as they are used by LMICs. https://secure-web.cisco.com/1pT7Rmm09awIYwPKI17ozYJcSE5dbA3GUpEEc5uaD6mGe5ito1XUEDRKCOXoZshSFyEpuVCz5WsBuotFciUIKPITDdlnNwHZEtulzwlKxJkAWq0KOgGpSmL8ix8f4BuWZkX3xzs4b1zNwDXOoWTKi4HQt1iFxBlHoUrUEKKJeNdqK7M9M9Ml4z1E5K4au_CK1yDm-

PWOOTC8zVSHgS_POmRgp9MA4herAgXHyHZOAhBe3GRiVmmOTigZGd6BY-zdV/https%3A%2F%2Fwww.who.int%2Fpublications%2Fi%2Fitem%2F9789241549912

Author response:
We thank the reviewer for this recommendation. We have added the current WHO ANC recommendations regarding utility of SFH now in the Background section of the manuscript (Lines 27-29).

4) To ensure consistency throughout the manuscript, suggest using the terminology "reference standards" instead of using gold and/or reference standard interchangeably.

Author response: Thank you for the suggestion. We have updated the manuscript to use the term "gold standard" consistently throughout to refer to the gold standard ultrasound comparison.

Reviewer #4: This manuscript addresses an important topic and is well written.

1. If the goal was to create a "standard" as it is put in one place in the methods, then the rationale for excluding studies with "strict eligibility criteria of optimally healthy populations within narrow BMI thresholds" is not clear. In fact, that is the population that should be included. Perhaps changing the wording or clarifying the objectives would help.

Author response: We apologize for the confusion. In the original version of the manuscript, we believe that we created confusion over our use of the words "norms" and "normative" in relation to SFH population reference data from unselected obstetric populations. We did not attempt to create a "standard" for optimal growth, as was done in the Intergrowth study.

Our objective was to evaluate and summarize symphysis fundal height (SFH) measurements for the purpose of gestational age assessment that can be applied in typical low-middle income country (LMIC) populations where optimal pregnancy health and fetal growth should not be assumed. Our goal was not to create a prescriptive "standard" for optimal size, like the Intergrowth study, as was assumed by reviewer #4 and the academic editor. We understand that the use of term 'normative' curves may have misled readers to interpret the curves as a 'standard,' which was not our intention. We have clarified the language in our revision.

We hope that we have clarified our aims in the revised manuscript and our response to the reviewers. In brief, INTERGROWTH-21st already established SFH standards based on assumptions of optimal fetal growth that can be used globally for fetal growth monitoring.

2. I am not familiar with the web-based WebPlotDigitizer application, as I'm sure potential readers might not be as well. Linear mixed models were later used for analysis. Perhaps some translation would help and/or I would defer to a statistician.

Author response: Thanks for the comment. WebPlotDigitizer is an open-source tool that has been shown to have high validity and reliability for extracting XY coordinates of plotted data (PMIDs: 27760807, 28268241, 27126988). The program has been used in systematic reviews (e.g., PMIDs: 32171442, 25512893) for papers that report continuous data in a chart without an accompanying table, in order to make the data included in the systematic review as comprehensive as possible.

3. The authors might add how the potential biases for study quality assessment were determined. I would think for example that training and following a standardized protocol would be important, but it's not clear to me if that criteria was assessed in the domains. Also this point is minor but it states "four" domains in line 76 but five are listed.

Author response: Details on study assessment are in the Appendix S1. We assessed study quality using the QUADAS-2 tool along four domains, but the addition of a comma in the manuscript that appeared to split the fourth domain into two separate components. In the original manuscript we reported that “Each study was evaluated for potential biases across four domains: patient selection, test method, reference standard, patient flow, and timing in pregnancy of SFH measurements.” The fourth domain should have read “and patient flow and timing in pregnancy of SFH measurements.” We have now clarified the domains with numbering in lines 88-89.

All of the individual assessment criteria within each of the four domains are reported in Appendix S1. We agree that quality assurance methods such as training, standardization, and supervision are important, and we assessed this under the “test method” domain.

4. I agree with the conclusions of the paper and think they are an important public health message. However, I imagine that there might always be places without access to ultrasound because they lack electricity or at least it will take time to remedy the lack of resources (having solar powered ultrasounds available for example). So before dismissing FH completely, the authors might discuss whether using customized fundal height (as championed by Gardosi) might better help diagnose preterm GA, and also the role of serial FH which seems to be an improvement over a single cross-sectional measurement. A longitudinal FH calculator has been developed (ref 1) and might be able to be applied for clinical use.

Author response: We agree that access to ultrasound will remain limited and realistically SFH will continue to be used in many settings to date pregnancies. We hope with this review to highlight the importance and urgency of expanding access to ultrasound for ANC given the wide prediction window around GA estimated by SFH. We’ve modified our discussion to clarify the two points that you’ve raised - that customized charts and modelling of serial SFH measurements can improve the accuracy of GA estimates somewhat. However, even with serial measurements, the accuracy of SFH-estimated GA had a prediction window of +/- 4 weeks in our systematic review.

5. This point is minor but the diagnostic accuracy was technically for "preterm" gestational age, not "preterm labor" (line 215).

Author response: Thank you for the comment. We have updated the manuscript to refer to preterm GA not preterm labor.

EDITOR COMMENTS:
The academic editor assessed the study and noted that the rationale for excluding studies from healthy populations is potentially problematic, and pointed out the potential for misleading interpretations to be drawn based on the emphasis on geography, particularly surrounding the discussion of "normative" curves. The academic editor noted that established uniformity of growth provides the rationale for international references curves such as INTERGROWTH-21, and that the discrepancy noted in the Asian studies is explained by higher occurrence of intrauterine growth restriction in South Asia, as compared to Africa.

AUTHOR RESPONSE:
Based upon reviewing the academic editor's feedback, we believe the main objective of the paper may have been misunderstood by the editors and have clarified the overall objective in our revision to Plos One.

Our objective was to evaluate and summarize symphysis fundal height (SFH) measurements for the purpose of gestational age assessment that can be applied in typical low-middle income country (LMIC) populations where optimal pregnancy health and fetal growth should not be assumed. Our goal was not to create a prescriptive "standard" for optimal size, like the Intergrowth study, as was assumed by reviewer #4 and the academic editor. We understand that the use of term 'normative' curves may have misled readers to interpret the curves as a 'standard,' which was not our intention. We have clarified the language in our revision. We have replaced 'normative' curve with 'population-based reference' throughout the paper, and hope that this clarifies the intent.

For the purpose of gestational age estimation in LMIC's, we excluded highly-selected cohorts from this study because these populations would not be representative of the general obstetric population. SFH measurements in these selected patients with only optimally healthy pregnancies would, on average, underestimate gestational age when applied to estimate gestational age in general obstetric populations where morbidities or fetal growth restriction are prevalent. For example, the Intergrowth study applied rigorous selection criteria that excluded pregnancies with socio-economic and health constraints on fetal growth, in order to demonstrate the optimal growth of fetuses. Intergrowth excluded women of low and high BMI, or with significant co-morbidities; these women comprise a large proportion of pregnancies in LMICs. Including studies conducted in only select, optimally healthy pregnancies would bias the relationship between SFH size and gestational age in a general obstetric population in an LMIC and therefore we intentionally excluded these studies.

We agree with the editor that the high prevalence of fetal growth restriction is the very reason for the lower mean SFH measurements in Asia. The relationship between SFH and gestational age in the healthy populations such as the Intergrowth study would not apply in the Asian setting where fetal growth restriction is prevalent, as demonstrated by our data. This is the reason that the reference SFH values from our study are clinically relevant and important for gestational age estimation in such settings when ultrasound/LMP are not available. Use of the Intergrowth SFH curve will systematically underestimate gestational age in South Asia.

References

1. Murad MH, Montori VM, Ioannidis JPA, Guyatt G, Rennie D, Meade MO, et al. Fixed-effects and random-effects models. Users' guide to the medical literature A manual for evidence-based clinical practice McGraw-Hill, 3rd ed New York, America. 2015;
